# A comparison between diuretics and angiotensin-receptor blocker agents in patients with stage I hypertension (PREVER-treatment trial): study protocol for a randomized double-blind controlled trial

**DOI:** 10.1186/1745-6215-12-53

**Published:** 2011-02-24

**Authors:** Flávio D Fuchs, Sandra C Fuchs, Leila B Moreira, Miguel Gus, Antônio C Nóbrega, Carlos E Poli-de-Figueiredo, Décio Mion, Luiz Bortolotto, Fernanda Consolim-Colombo, Fernando Nobre, Eduardo Barbosa Coelho, José F Vilela-Martin, Heitor Moreno, Evandro José Cesarino, Roberto Franco, Andréa Araujo Brandão, Marcos R de Sousa, Antônio Luiz Pinho Ribeiro, Paulo Cesar Jardim, Abrahão Afiune Neto, Luiz César N Scala, Marco Mota, Hilton Chaves, João Guilherme Alves, Dario C Sobral Filho, Ricardo Pereira e Silva, José A Figueiredo Neto, Maria Cláudia Irigoyen, Iran Castro, André Avelino Steffens, Rosane Schlatter, Renato Bandeira de Mello, Francisca Mosele, Flávia Ghizzoni, Otávio Berwanger

**Affiliations:** 1 Hospital de Clínicas de Porto Alegre, Universidade Federal do Rio Grande do Sul, Porto Alegre, Brazil; 2 Hospital Universitário Antônio Pedro, Universidade Federal Fluminense, Niterói, Brazil; 3 Hospital São Lucas, Pontifícia Universidade Católica do Rio Grande do Sul, Porto Alegre, Brazil; 4 Hospital das Clinicas, Universidade de São Paulo, São Paulo, Brazil; 5 Instituto do Coração, Universidade de São Paulo, São Paulo, Brazil; 6 Faculdade de Medicina de Ribeirão Preto - Universidade de São Paulo, Ribeirão Preto, Brazil; 7 Faculdade de Medicina São José do Rio Preto, São José do Rio Preto, Brazil; 8 Faculdade de Ciências Médicas, Universidade de Campinas, Campinas, Brazil; 9 Faculdade de Ciências Farmacêuticas, Universidade de São Paulo, Ribeirão Preto, Brazil; 10 Faculdade de Medicina de Botucatu, Universidade Estadual de São Paulo, Botucatu, Brazil; 11 Universidade do Estado do Rio de Janeiro, Rio de Janeiro, Brazil; 12 Hospital das Clínicas, Universidade Federal de Minas Gerais, Belo Horizonte, Brazil; 13 Hospital das Clínicas de Goiânia, Universidade Federal de Goiás, Goiânia, Brazil; 14 Anis Rassi Hospital, Goiânia, Brazil; 15 Hospital Universitário Júlio Muller, Universidade Federal de Mato Grosso, Cuiabá, Brazil; 16 Faculdade de Medicina, Universidade de Ciências da Saúde Alagoas, Maceió, Brazil; 17 Faculdade de Medicina, Universidade Federal de Pernambuco, Recife, Brazil; 18 Instituto de Medicina Integral Prof Fernando Figueira, Recife, Brazil; 19 Hospital Universitário Oswaldo Cruz/PROCAPE, Universidade de Pernambuco, Recife, Brazil; 20 Hospital Universitário Valter Cantídio, Universidade Federal do Ceará, Fortaleza, Brazil; 21 Hospital Universitário, Universidade Federal Maranhão, São Luiz, Brazil; 22 Instituto de Cardiologia, Fundação Universitária de Cardiologia, Porto Alegre, Brazil; 23 Faculdade de Medicina, Universidade Federal de Pelotas, Pelotas, Brazil; 24 Hospital do Coração, São Paulo, Brazil

## Abstract

**Background:**

Cardiovascular disease is the leading cause of death in Brazil, and hypertension is its major risk factor. The benefit of its drug treatment to prevent major cardiovascular events was consistently demonstrated. Angiotensin-receptor blockers (ARB) have been the preferential drugs in the management of hypertension worldwide, despite the absence of any consistent evidence of advantage over older agents, and the concern that they may be associated with lower renal protection and risk for cancer. Diuretics are as efficacious as other agents, are well tolerated, have longer duration of action and low cost, but have been scarcely compared with ARBs. A study comparing diuretic and ARB is therefore warranted.

**Methods/design:**

This is a randomized, double-blind, clinical trial, comparing the association of chlorthalidone and amiloride with losartan as first drug option in patients aged 30 to 70 years, with stage I hypertension. The primary outcomes will be variation of blood pressure by time, adverse events and development or worsening of microalbuminuria and of left ventricular hypertrophy in the EKG. The secondary outcomes will be fatal or non-fatal cardiovascular events: myocardial infarction, stroke, heart failure, evidence of new subclinical atherosclerosis and sudden death. The study will last 18 months. The sample size will be of 1200 participants for group in order to confer enough power to test for all primary outcomes. The project was approved by the Ethics committee of each participating institution.

**Discussion:**

The putative pleiotropic effects of ARB agents, particularly renal protection, have been disputed, and they have been scarcely compared with diuretics in large clinical trials, despite that they have been at least as efficacious as newer agents in managing hypertension. Even if the null hypothesis is not rejected, the information will be useful for health care policy to treat hypertension in Brazil.

**Clinical trials registration number:**

ClinicalTrials.gov: NCT00971165

## Background

Cardiovascular disease (CVD) is the leading cause of death in Brazil, and high blood pressure is its major risk. factor. The prevalence of hypertension in Brazil is within 22.3 to 44% of adults [1]. The benefit of drug treatment of hypertension to prevent major cardiovascular events was consistently demonstrated in a large series of clinical trials controlled by placebo. The superiority of any particular agent among the groups of blood pressure-lowering drugs was investigated in various clinical trials. ALLHAT, the largest and better designed trial, showed that chlorthalidone had similar efficacy to prevent fatal and non-fatal coronary events as an ACE inhibitor (lisinopril) and a calcium channel blocker agent (amlodipine) [2]. Chlorthalidone was superior to lisinopril in the prevention of other cardiovascular outcomes, particularly of stroke in black participants, and it was superior to amlodipine in the prevention of heart failure. In the VALUE trial, amlodipine was superior to valsartan, an angiotensin-receptor blocker (ARB) agent, in the prevention of CHD and stroke [3]. The most recent and extensive meta-analysis of trials that compared the efficacy of blood pressure-lowering drugs against placebo and against each other failed to demonstrated substantial advantage of any group of agents [4]. Nonetheless, ARBs have been preferential drugs in the management of hypertension, being five out of the ten agents more frequently prescribed in the USA in 2007 [5].

Data from some trials have shed some concern about the safety of ARB agents [6], but these findings require corroboration in other studies. The main concern is with the guidelines' supported preference for ARB agents to prevent renal damage, particularly in patients with diabetes. Most data supporting such preference came from placebo controlled trials, not controlling for their blood pressure-lowering effect. These agents did not show any superiority over angiotensin converting enzyme (ACE) inhibitors to prevent renal outcomes, and the association of these agents was clearly deleterious in the ONTARGET trial [7]. It is of note that even the beneficial effects of ACE inhibitors in this context were mostly demonstrated in studies not controlled by other blood pressure agents. In the only study that compared an ACE inhibitor with a diuretic, the incidence of microalbuminuria was similar [8]. In the ALLHAT trial, the incidence of end-stage renal disease was about 70% higher in patients with diabetes and with glomerular filtration rate between 60 and 80 ml/min, randomized to lisinopril and anlodipine, instead of chlorthalidone [9].

A complex clinical trial investigated the efficacy of an ARB agent and of an ACE inhibitor to prevent renal damage in patients with type I diabetes [10]. Change in mesangial fractional volume per glomerulus over the 5-year period of follow-up, the primary outcome, did not differ significantly between the placebo and treatment groups. Moreover, the 5-year cumulative incidence of microalbuminuria was 17% with losartan versus 6% with placebo and 4% with enalapril (P = 0.01).

There is no head-to-head comparison between diuretics and ARB agents in the prevention of hard cardiovascular outcomes, and even the comparison of their blood pressure-lowering effects was scarcely reported. Despite this, ARB agents are the leading brands in terms of profits in various countries in the world, including Brazil. This leadership seems to be based on a strong commercial strategy, which may have introduced bias in the evidences of clinical trials in favor of these drugs [11,12]. The idea that they have blood pressure-independent effects is accepted by most, despite the evidences of better designed trials and of the powerful meta-analysis of Law, Morris and Wald [4]. The option of an ARB agent, instead of a diuretic, as the first line approach in the public health system in Brazil would result in a large expenditure of resources, and there is a pressure to include them in the list of essential drugs provided by the government.

Diuretics are at least as efficacious as other blood pressure-lowering drugs, are well tolerated, have longer duration of action and the advantage of very low cost to be used in a population intervention [13]. Chlorthalidone is a more efficacious agent, and should be the preferential choice of diuretic to be employed in clinical practice [13]. Its main limitation is to induce hypokalemia in some patients, an adverse effect that can be antagonized by a potassium-sparing diuretic, such as amiloride [14].

A study comparing diuretic with an ARB agent is therefore scientifically required. It would be also important in Brazil, in order to support the decisions of the Public Health System in regard to blood pressure drugs supply for the Brazilian population. Such a study was demanded and funded by the Health and Technology Ministries in Brazil.

## Trial rationale

ARB agents have good tolerability and putative pleiotropic effects, particularly renal protection, which support their preference by physicians as an antihypertensive agent. Independent assessments of the evidence have questioned these advantages, and a concern with the safety of these agents, such as the loss of nephroprotection in patients with diabetes, has emerged. Diuretics, particularly chlorthalidone, are as efficacious as newer agents in managing hypertension. Its main adverse effect, hypokalemia, which is associated with arrhythmias and hyperglycemia, can be circumvented by the association of potassium-sparing diuretic, such as amiloride. Blood pressure has been a valid surrogate of the beneficial effects of blood pressure-lowering drugs. In this scenario, the PREVER treatment trial will compare the antihypertensive efficacy of the association of chlorthalidone plus amiloride with losartan, for the initial management of hypertension (Clinical trials registration number: NCT00971165). Other intermediate outcomes, such as microalbuminuria and left ventricular hypertrophy, and major cardiovascular outcomes, are going to be investigated. Even if the null hypothesis is not rejected (diuretics equivalent to losartan), the information will be useful for health care policy to treat hypertension in Brazil.

## Research questions

1. Is losartan more efficacious and safe than the association of chlorthalidone with amiloride as the first option to control blood pressure in patients with stage I hypertension?

2. Is losartan more efficacious than the association of chlorthalidone with amiloride as the first option to prevent the development of target-organ damage in patients with stage I hypertension?

3. Is losartan more efficacious than the association of chlorthalidone with amiloride as the first option to prevent the occurrence of major cardiovascular events in patients with stage I hypertension?

## Methods

### Design

randomized, double-blind, clinical trial, controlled by an active treatment.

### Eligible participants

patients older than 40 years of age with Stage I hypertension.

### Exclusion criteria

low life expectancy, other indications for the use of diuretics, such as cardiovascular disease, intolerance to the study drugs, pregnancy.

### Random allocation

by a computer generated list, using a validated software (Random allocator), with variable block sizes and stratified by center.

### Interventions

**Figure 1:**
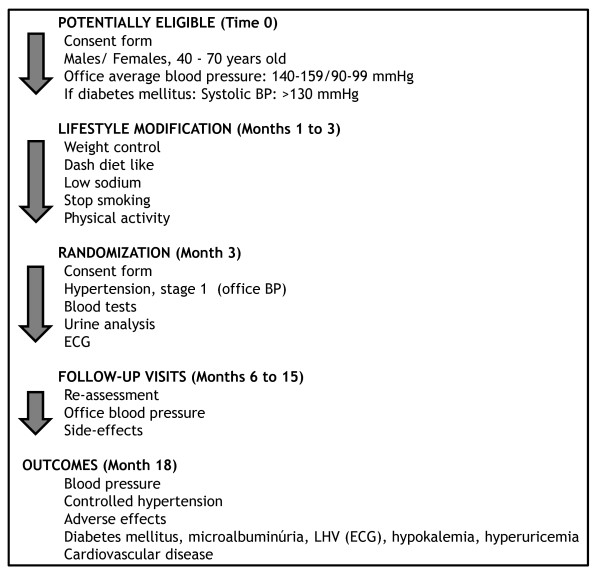
**Flow chart of the PREVER-Treatment trial, describing the selection, randomization and follow-up process**.

Chlorthalidone plus amiloride up to 25 and 5 mg daily, versus losartan up to 100 mg daily. Amlodipine up to 10 mg daily and propranolol up to 160 mg/day, in an open fashion, will be added if blood pressure is not controlled. Figure 1 shows flow-chart of selection of participants and interventions.

### Outcomes

#### Primary

1. Blood pressure variation and proportion of use of add-on drugs.

2. Adverse events.

3. Development or worsening of microalbuminuria and of left ventricular hypertrophy in the EKG.

#### Secondary

fatal or major cardiovascular events.

### Follow-up and duration of the study: outpatient clinical visits

**Figure 2:**
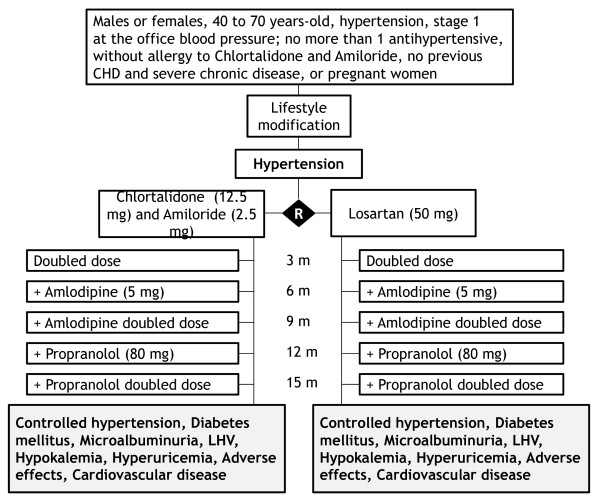
**Summary of the PREVER-Treatment trial key practical aspects**.

for evaluation and enrollment and thereafter consultations at the 3th., 6th., 9^th^, 12^th^. and 18^th^. months. Figure 2 shows the summary of key practical aspects of the trial.

### Assessment of outcomes

Blood pressure will be measured at the follow-up visits by average blood pressure (two measurements by an automatic electronic device Microlife BP 3BTO-A, licensed for fabrication by Micromed Biotecnologia Ltda, Brasília, Brazil).

#### Adverse events

adverse events will be investigated by open questions and by a semi-structured questionnaire including general symptoms and the presumed adverse effects of the drugs used in the trial. Laboratory adverse events, such as hypokalemia, hyperuricemia and diabetes (glycated hemoglobin and fasting glucose) will be investigated at the final visit of the participants

#### Target-organ damage

Microalbuminuria will be determined by nephelometry. Left ventricular hypertrophy will be investigated by electrocardiogram, using the Sokolow-Lyon voltage and the Cornell voltage-duration product.

#### Cardiovascular outcomes

**Figure 3:**
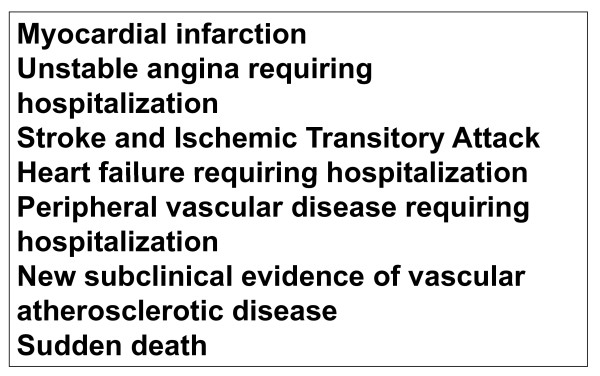
**PREVER-Treatment trial cardiovascular outcomes**.

from a statistical point of view they will be secondary events, in face of the power of the sample calculated for the trial. Figure 3 presents the main outcomes that will be investigated. The cases will be adjudicated on the basis of interview, hospital charts and exams, death certificates and verbal autopsy with next-of-kin, by members of the outcome committee.

### Sample size calculation

for non-inferiority, with a P alpha of 0.01, power of 99%, and SD of 12 mmHg, and maximum acceptable absolute difference of 4 mmHg (diastolic), it will be necessary to study 433 patients per group, rounded to 500 patients to compensate losses. The difference of 5 mmHg (diastolic) has been achieved, on average, in clinical trials that have demonstrated the advantage of a drug over placebo or other drug in the prevention of major cardiovascular outcomes. In face of the other primary hypotheses of the trial, and other subprojects that will be undertaken in some centers, it was decided to study 1200 patients per group.

### Statistics

the cumulative incidence of target-organ damage and adverse events will be analyzed by Chi-square test. Blood pressure will be compared by ANOVA for multiple measurements and factors, testing for the time-group interaction. The primary analysis will be by intention-to-treat. An attempt to measure blood pressure at the end of trial even for participants that abandoned the treatment will be undertaken. The rates of controlled blood pressure by treatment will be evaluated by survival curves and tested by Cox models, censoring the cases lost in the follow-up. In case of uneven losses by group, sensitivity analysis will be undertaken.

### Logistics

This is a nation-based trial, with 24 clinical centers distributed in 9 States. A Coordinating Committee is responsible for the elaboration of this proposal and for the main decisions of the trial. The study organizational chart will include an executive Committee, a safety committee, outcome committee, lab and EKG centers, and the research units.

## Ethical approval

The project and the informed consent form were approved by the Ethics committee of the Hospital de Clínicas de Porto Alegre, which is accredited by the Office of Human Research Protections as an Institutional Review Board, and by the Ethic Committees of the participating centers. All participants will be asked to sign the informed consent to participate in the study

## Abbreviations

ACE: angiotensin converting enzyme; ALLHAT: The Antihypertensive and Lipid-Lowering Treatment to Prevent Heart Attack Trial; ARB: Angiotensin-receptor blockers; CHD: coronary heart disease; CVD: Cardiovascular disease; EKG: electrocardiogram; ONTARGET: The Ongoing Telmisartan Alone and in Combination with Ramipril Global Endpoint Trial; PREVER: Prevention of cardiovascular events in patients with hypertension and pre-hypertension study; VALUE: Valsartan Antihypertensive Long-term Use Evaluation

## Competing interests

The authors declare that they have no competing interests.

## Authors' contributions

FDF conceived the study; all authors participated in the trial design and methodological considerations, contributed to the draft of this manuscript for intellectual content and approved its final version. They are the coordinators of the clinical centers that will enroll the trial participants.
